# Intangible cultural heritage as school-based psychosocial support for left-behind adolescents in rural China: a framework and preliminary pilot study

**DOI:** 10.3389/fpsyt.2026.1899748

**Published:** 2026-08-17

**Authors:** Yichen Li, Lidong Wang

**Affiliations:** 1 College of Art, Southwest Minzu University, Chengdu, Sichuan, China; 2 Design Academy, Sichuan Fine Arts Institute, Chongqing, China

**Keywords:** art therapy, cultural identity, emotion, intangible cultural heritage, language, mental health, motivation, rural adolescents

## Abstract

Rural left-behind adolescents in China experience elevated risks of loneliness, negative affect, weakened belonging, and limited access to acceptable psychosocial support. Conventional school counselling models often depend on self-referral, verbal disclosure, and identification with a help-seeking role, conditions that may not fit the social and cultural context of many rural students. This manuscript develops and preliminarily evaluates a culturally grounded schoolbased psychosocial support programme using locally rooted intangible cultural heritage (ICH) activities, including paper cutting, embroidery, local song, and related symbolic practices.

This study is a preliminary non-randomized waitlist-controlled pilot study with theoretical framework development and embedded qualitative process data. The programme was not art therapy in the formal clinical or professional sense: it was not delivered by credentialed art therapists, did not involve psychotherapeutic interpretation of artwork, and was not intended as treatment for diagnosable mental disorders. It is therefore conceptualized as an ICH-based arts-and-health psychosocial support programme for subclinical wellbeing needs in a rural school context.

The proposed Motivation–Emotion–Language framework integrates self-determination theory, emotion regulation research, and Vygotskian cultural mediation. In a rural lower-secondary school in southwestern China, 28 left-behind students participated in a six-week ICH programme and 24 comparable students served as a waitlist comparison group. Compared with the waitlist group, the ICH group showed greater pre-post improvement in loneliness (*d* = 0.58, 95% CI [0.21, 0.95], *p* = .002), negative affect (*d* = 0.45, 95% CI [0.09, 0.81], *p* = .014), cultural identity (*d* = 0.53, 95% CI [0.17, 0.89], *p* = .004), and school belonging (*d* = 0.36, 95% CI [0.01, 0.71], *p* = .044). Because the study used a small non-randomized waitlist-controlled design, these findings should be interpreted as preliminary estimates rather than definitive evidence of efficacy.

Illustrative qualitative process data suggested that students often engaged first through making rather than verbal disclosure, and that local motifs and songs elicited recognition of shared cultural belonging. Findings should be interpreted cautiously because of the small sample, nonrandomized allocation, short intervention duration, waitlist rather than active-control design, and adaptive introduction of singing activities in the final sessions. The study provides preliminary feasibility and outcome signals rather than definitive evidence of efficacy or mechanism. Future research should use randomized controlled trials, active comparison groups, longitudinal follow-up, repeated process measurement, mediation analyses, and implementation research.

## 1 Introduction

Rural adolescent mental health in China presents both a service gap and a cultural-fit problem. Although school-based psychological services have expanded in recent years, many rural adolescents remain unlikely to use conventional counselling resources. This is particularly true for left-behind adolescents, whose parents migrate for work and who often live for extended periods with grandparents or other relatives. For these students, distress may be expressed through withdrawal, loneliness, academic disengagement, irritability, or bodily complaints rather than through direct requests for psychological help. A support model that depends on self-referral, verbal disclosure, and willingness to occupy a “student with problems” role may therefore be formally available but practically inaccessible.

The scale of the left-behind child phenomenon gives this issue substantial public health and educational significance. Rural-to-urban labour migration has left many children and adolescents growing up with one or both parents absent for long periods of the school year. Previous studies have shown that left-behind children are at increased risk of loneliness, depressive symptoms, anxiety, school disengagement, and reduced socioemotional wellbeing compared with peers living with non-migrant parents (1–3). Age at separation and quality of substitute caregiving are important moderators: parental absence does not determine poor outcomes, and many left-behind children show resilience, but the pattern of elevated psychosocial risk is sufficiently consistent to require school and community responses.

Schools are the most accessible setting for preventive and supportive work. In many rural counties, schools have established counselling rooms, designated mental health teachers, or basic psychological education activities. These developments are important, but they do not fully resolve the access problem. In low-resource schools, a single teacher may be responsible for hundreds of students and may have limited specialist training or supervision. More fundamentally, students must often identify themselves as needing psychological help before support begins. This requirement can be a barrier in communities where emotional disclosure is stigmatized, where young people are encouraged to endure hardship quietly, and where help-seeking may be perceived as burdening the family. Although more empirical data are needed on rural adolescents’ actual use of counselling rooms, existing evidence on stigma, rural service shortages, and low help-seeking in youth mental health suggests that service availability alone does not ensure engagement.

This paper asks whether psychosocial support can be delivered through a medium that lowers these barriers. Specifically, it considers whether locally rooted intangible cultural heritage (ICH) practices can provide an acceptable and culturally meaningful route into school-based wellbeing support. ICH practices such as paper cutting, embroidery, local songs and ballads, shadow puppetry, festival objects, clay modelling, and regional performance traditions are often inexpensive, locally recognizable, and embedded in intergenerational community life. A student who cuts a paper pattern, stitches a familiar motif, or hears a local song is not necessarily entering a counselling session; they are participating in a cultural activity that may already carry family and community meaning. This difference in how support is encountered may be psychologically important.

The present study draws on several related bodies of literature. The arts-and-health literature has documented associations between arts participation and improved health and wellbeing across varied populations and settings (4–6). Expressive arts and art therapy literatures further suggest that making, image, sound, movement, and material form can support emotional expression and regulation, especially when verbal disclosure is difficult (7–10). However, the programme described in this manuscript should not be understood as art therapy in the formal clinical or professional sense. It was not delivered by credentialed art therapists, did not use psychotherapeutic interpretation of participants’ artwork, and was not designed as treatment for diagnosable mental disorders. It is more accurately situated within the broader fields of arts and health, culturally responsive psychosocial support, community arts, and school-based wellbeing promotion.

A second relevant literature concerns culturally adapted mental health and psychosocial interventions. Cultural adaptation is not simply a matter of translating language or adding local examples to an imported programme. It requires attention to how distress is expressed, which forms of help are acceptable, who is trusted to provide support, and what symbolic resources are already meaningful in the community. For rural Chinese adolescents, especially left-behind students, interventions that begin from local cultural participation may avoid some of the stigma and verbal demands associated with conventional counselling. At the same time, such interventions should not be romanticized. They may work through general factors common to many supportive group activities, including adult attention, peer contact, novelty, skill-building, and enjoyable shared activity. Whether local cultural specificity contributes additional benefit remains an empirical question.

The third literature concerns intangible cultural heritage itself. UNESCO’s Convention for the Safeguarding of the Intangible Cultural Heritage (11) emphasizes transmission, continuity, and community participation. In China, school-based ICH transmission has become an important cultural policy goal. Yet the psychological and developmental functions of such programmes remain underexamined. Most ICH education is discussed in terms of cultural preservation, aesthetic education, or local identity, rather than as a potentially supportive context for adolescent wellbeing. This manuscript therefore sits at the intersection of school mental health, arts and health, cultural participation, and ICH transmission.

The paper has two aims. The first is conceptual: to develop a Motivation–Emotion–Language framework explaining how locally rooted ICH participation may support rural adolescents’ psychosocial wellbeing. The framework integrates self-determination theory (12–14), emotion regulation research (15, 16), and Vygotskian cultural mediation (17). It proposes that ICH activities may support wellbeing by increasing voluntary engagement and competence, enabling nonverbal emotional expression and regulation, and reconnecting students with local language, motif, narrative, and cultural identity.

The second aim is empirical but preliminary: to report a small non-randomized waitlist-controlled pilot study of a six-week ICH-based psychosocial support programme for left-behind adolescents in a rural school in southwestern China. The study examines feasibility, attendance, preliminary pre-post outcome signals, and illustrative qualitative process data. It does not provide a definitive test of efficacy, and it does not directly test the proposed mechanisms. In particular, the design cannot determine whether observed changes are attributable to the local cultural specificity of ICH rather than to general features of structured group activity. The pilot should therefore be interpreted as an early step toward more rigorous active-control and longitudinal research.

## 2 The rural mental health gap and the left-behind condition

### 2.1 Developmental and relational foundations

Attachment theory provides a useful framework for understanding the developmental significance of long-term parental absence. Bowlby’s (18) account holds that children construct internal working models of relationships through accumulated experience with caregivers who are available, responsive, and consistent over time. These models are shaped by the density and quality of ordinary daily interaction: being responded to, corrected, comforted, and accompanied through routine life. Long parental absences do not automatically produce insecure attachment, and many grandparents provide warm and reliable care. However, migration can reduce the density of parent-child interaction, and this reduction may accumulate across years in ways that are difficult for young people to name.

Belonging compounds the picture. Baumeister and Leary’s (19) account of belongingness as a fundamental human motivation describes a need that left-behind adolescents may experience as fragile in daily life. Many such students understand and respect their parents’ economic sacrifice, yet may also feel resentment, guilt, loneliness, or shame. These layered feelings can be difficult to articulate, especially in households where hardship is normalized and where expressing emotional need is perceived as adding to adult burdens. Children in such households may learn that certain feelings should be managed privately rather than spoken.

Secondary losses can compound the primary loss of everyday parental presence. An absent parent cannot consistently attend school events, help navigate peer conflicts, supervise homework, or provide daily emotional modelling. Substitute caregivers may be devoted, but they may have limited formal education, limited familiarity with adolescent development, and substantial agricultural or family responsibilities. When academic difficulties develop alongside emotional difficulties, students may disengage from school, losing a structure that might otherwise provide routine, peer contact, and adult recognition.

### 2.2 Resilience and protective factors

This account should not be read as a deficit model of left-behind children. Resilience research is clear that adversity does not predict poor outcomes when adequate protective factors are present (20–22). Protective factors include reliable adult relationships, domains of recognized competence, stable peer belonging, meaningful roles in a group or community, and participation in something larger than the self. These resources do not remove the structural cause of parental migration, but school-level intervention can help create or strengthen them.

Bronfenbrenner’s ecological systems theory (1979) helps locate these resources. Development takes place within nested systems: family, school, peer group, community, and broader culture. When the family microsystem is thinned by migration, other systems can partly compensate if they provide stable adult relationships, structured roles, shared activities, and cultural anchors. Local ICH practices are situated at the intersection of school, community, and culture. They are accessible through school, rooted in community, and carry symbolic and narrative material that may support continuity and belonging.

Antonovsky’s (23) sense of coherence adds a related dimension. The capacity to experience one’s life as comprehensible, manageable, and meaningful is protective across adversities. Cultural practices may contribute to such coherence by providing stable symbols, seasonal rhythms, inherited narratives, and collective participation that make individual experience legible within a wider pattern. For adolescents whose family lives are marked by absence and uncertainty, reconnection with stable cultural forms may help restore a sense of continuity and place.

### 2.3 The structural mismatch

The rural mental health gap is therefore not only a shortage of clinicians. It is also a gap in form, access, and cultural fit. A service model premised on voluntary self-referral, verbal emotional disclosure, and a designated help-seeking role does not match how many rural adolescents experience and manage distress. Ryff’s (24) multidimensional account of psychological wellbeing emphasizes positive relations, purpose, and personal growth alongside the absence of symptoms; addressing wellbeing in this fuller sense requires more than a counselling room staffed by an overburdened teacher.

What is needed is a medium that is available in the school and community, does not require selfidentification as troubled, allows feeling to be held and worked with before it is spoken, and is experienced as belonging to local life rather than imported from elsewhere. Rural ICH practices may meet these criteria in ways that generic activities or imported techniques do not, although this claim requires careful empirical testing.

## 3 Intangible cultural heritage as a medium: a motivation, emotion, language framework

### 3.1 ICH as a mediating cultural resource

The theoretical starting point is Vygotsky’s account of cultural tools and mediation (17). Higher mental functions develop through engagement with culturally organized artifacts: language, material forms, symbolic systems, performance conventions, and patterned practices embedded in collective life. A paper-cut motif, for example, is not only a physical form. It may signify reunion, protection, seasonal transition, or communal blessing in ways that are legible to those who share the cultural context.

This distinction is central to the present argument. A blank sheet of paper asks a student to invent meaning from nothing. A red sheet of paper used for a familiar window-flower pattern offers structure, legitimacy, symbolic vocabulary, and a route into participation that does not require personal exposure at the outset. The inherited form provides both cover and scaffolding. The student can make something meaningful without announcing that it is personally meaningful. For adolescents who are guarded or unused to emotional disclosure, this indirection may make participation possible.

The self-construal literature is relevant here (25). In more interdependent cultural contexts, selfhood is constituted partly through relational membership and group participation rather than exclusively through private introspection. A craft or performance practice rooted in local tradition may therefore be a culturally fitting vehicle for self-expression not because it is less personal, but because it is personal through shared cultural membership.

### 3.2 What specific ICH forms afford

Different ICH forms make different kinds of participation possible, and these differences matter for programme design. Paper cutting is often an accessible entry point. Materials are inexpensive, a satisfying product can be completed within one session, and traditional motifs carry recognized meanings such as reunion, protection, abundance, and seasonal transition. Beginners can copy simple symmetrical patterns, while personal variation can emerge as skill develops.

Embroidery works differently. It is slower and requires sustained attention. The repeated movement of needle and thread and the gradual accumulation of colour and form may support concentration and calm. Embroidery has also historically been a social practice, often done in proximity to others, and this social quality is relevant to small-group settings in which connection can develop quietly through shared work.

Folk song and local ballad introduce dialect, place, and oral narrative. A local song may carry references to local landscape, seasonal practices, family memory, or community events. Such material may connect adolescents to grandparents, household memory, and pre-migration domestic worlds. Because singing requires being heard, these forms may be better introduced after a group has developed safety through making-based activity.

Shadow puppetry and regional opera are inherently collective forms. They involve scripts, roles, coordination, rehearsal, and performance. Their value may be strongest in later stages of a programme, when students are ready to take collective risks and when the group has sufficient cohesion to sustain a shared task. Clay and figure modelling provide tactile and largely nonverbal expression. The material’s malleability is psychologically important: a closed shape can be opened, a solitary figure can be given companions, and a heavy form can be reworked into something lighter.

**Table 1** summarizes these forms across dimensions relevant to programme planning.

**Table 1:** Common ICH forms compared by initial accessibility, cost and safety, proposed layer emphasis, and suggested programme stage.

Form	Initial accessibility	Cost and safety	Proposed layer emphasis	Suggested stage
Paper cutting	Low threshold	Very low; safe with supervision	Motivation and emotion	Early
Clay sculpture	Low threshold	Low; safe	Emotion	Early
Embroidery	Low to moderate threshold	Low; safe with supervision	Emotion and sustained attention	Early to middle
Folk song and ballad	Moderate threshold	Very low; safe	Language and identity	Middle
Shadow puppetry	High threshold	Moderate; requires supervision	Language and relatedness	Late
Regional opera	High threshold	Moderate to high	Language and relatedness	Late

These classifications are practice-based planning categories rather than empirically validated rankings. They are based on material cost, safety, technical complexity, time required to complete a satisfying product, and the degree of collective coordination required. They should be refined in future implementation research.

### 3.3 Motivation: the entry condition

Motivation is the entry condition for the framework. If students do not attend voluntarily or disengage after initial sessions, no emotional or identity-related process can develop. Self-determination theory (12–14) identifies three basic psychological needs: competence, autonomy, and relatedness. Activities that satisfy these needs are more likely to support voluntary engagement.

ICH craft may address competence in a direct and immediate way. A paper cut completed in one session can be held, examined, shown to peers or family members, and taken home. This differs from many school tasks, where success is deferred, externally evaluated, or uncertain. When challenge and skill are well matched, students may enter absorbed engagement similar to the state Csikszentmihalyi (26) describes as flow.

Autonomy depends on genuine choice within structure: which motif to use, which colour arrangement to select, whether to follow a traditional pattern closely or introduce variation, and whether to connect the work to a personal memory or festival occasion. A supportive ICH group should provide enough scaffolding to make participation possible for beginners and enough openness to preserve students’ sense that their choices are their own.

Relatedness is where locally rooted ICH may offer an advantage over generic craft. Many ICH forms were historically social: embroidery done in groups, songs sung in call and response, festival objects prepared collectively, and performances that gathered villages. In a school setting, peer connection can develop through shared work without requiring students to announce loneliness or make direct interpersonal demands. Connection grows sideways through the activity.

The presence of a local heritage bearer may add an intergenerational dimension. The bearer represents a living connection to community practice and cultural memory. For left-behind adolescents whose family intergenerational relationships may be attenuated by migration, learning a valued skill from a skilled elder may carry relational significance beyond technical instruction.

### 3.4 Emotion: expression before articulation

Once students are attending and engaged, ICH activities may provide routes for emotional expression and regulation that do not depend on verbal disclosure. Gross’s process model of emotion regulation (15, 16) identifies strategies that operate through situation selection, attention, appraisal, and response modulation. Talk-based counselling often targets appraisal and verbal reflection. For adolescents who cannot or will not label and discuss emotional states, additional routes may be needed.

The first route is nonverbal externalization. Expressive arts and art therapy literatures suggest that feelings can be given form in objects, images, sound, and movement before they take linguistic shape (8, 9). A student may cut, stitch, press clay, or hold a puppet in ways that reflect an emotional state without naming it. The made object is both connected to the student and outside them: visible, handleable, modifiable, and shareable on the student’s terms.

The second route is the regulatory quality of making. Repetitive rhythmic manual activity, such as folding and cutting, stitching, or shaping clay, may support attention and reduce perceived arousal. Kaimal and colleagues (27) reported reductions in cortisol following art-making, although such findings should not be overgeneralized across populations and contexts. The practical point is that students may experience the activity as calming, and this subjective calm is relevant to psychosocial support.

The third route is symbolic reworking. Once an emotional state has been externalized into an object, the object can be changed. A solitary clay figure can be given companions, a closed paper form can be opened, and a dense pattern can be relieved by lighter colours. These transformations are analogous to reappraisal in that they alter meaning, but they operate through material and image rather than through explicit verbal analysis.

### 3.5 Language: cultural re-anchoring and identity

The third layer distinguishes the framework from a general account of group craft. It concerns the role of dialect, oral narrative, motif, and visual symbolic vocabulary in adolescent identity and belonging.

Bruner’s (28) account of narrative selfhood is relevant here. People organize experience into stories that give it coherence, continuity, and meaning. Local oral traditions, festival narratives, ballads, and stories attached to motifs offer inherited narrative structures into which young people can place experience. These structures are carried in local language, sound, seasonal associations, and familial transmission chains that may be emotionally familiar before they are analytically understood.

For left-behind students, reconnection to local narrative and linguistic material is not simply nostalgia. It may re-anchor identity in something that persists despite parental absence. The family has been disrupted; the community’s cultural forms remain present in grandparents’ households, local festivals, and the knowledge of heritage bearers. Reconnecting with them in school may allow students to claim membership in a cultural community that predates and exceeds the immediate family.

The visual symbolic vocabulary of ICH forms functions similarly. Paper-cut motifs, embroidery patterns, puppet characters, and festival objects carry meanings around reunion, protection, longevity, seasonal transition, and communal aspiration. Learning to use that vocabulary is a form of cultural literacy acquired through participation, imitation, and social recognition. Social identity theory (29) and research on ethnic and cultural identity (30, 31) suggest that secure group identification can contribute to psychological wellbeing, particularly in adolescence. Erikson’s (32) account of identity formation as a central adolescent task gives this a developmental frame.

### 3.6 The integrated framework

**Table 2** summarizes the proposed framework.

**Table 2:** Proposed motivation–emotion–language framework.

Layer	Theoretical basis	Proposed mechanism	Expected time course	Expected proximal outcome
Motivation	Self-determination theory	Craft satisfies needs for competence, autonomy, and relatedness; cultural familiarity lowers participation threshold	Immediate; supports attendance across sessions	Voluntary and sustained participation
Emotion	Emotion regulation theory; expressive arts literature	Nonverbal externalization, rhythmic calming through making, and symbolic reworking of emotional material	Within and across sessions	Reduced negative affect and perceived distress
Language	Narrative theory; cultural identity research; Vygotskian mediation	Dialect, oral narrative, and visual motif re-anchor identity and belonging in locally recognized cultural material	Potentially slower and cumulative	Strengthened cultural identity, belonging, and self-worth

The mechanisms and time course shown in this table are theoretical propositions. The present pilot study was not designed to test mediation or the temporal ordering among the three layers.

The layers are not independent. Motivation is the condition under which emotional work becomes possible. Sustained emotional engagement may then create conditions for identity-relevant shifts. This ordering is a testable prediction, not an established sequence. It requires session-by-session measurement and mediation analysis that were not included in the present pilot.

### 3.7 The question of locality

The most important objection to the framework is that its active ingredients may be general: competence experience, peer contact, warm adult attention, novelty, and structured enjoyable activity. If this is correct, any well-run group craft programme might produce similar results, and local cultural specificity would be incidental.

This objection is central rather than peripheral. General factors are certainly present in the model and may contribute substantially. The specific claim is that local ICH may add an identity and belonging layer through symbolic and narrative content recognized by the students’ own community. A folk song from another province may be enjoyable as music, but it may not carry the same relationship to a student’s household memory, festival calendar, and intergenerational belonging. A craft tradition imported from elsewhere may build skill, but it may not provide the inherited symbolic vocabulary through which local membership is recognized. This claim requires empirical testing through active-control designs comparing local ICH programmes with generic or non-local craft programmes of equivalent structure, duration, adult attention, and group contact.

## 4 Methods and practice model

### 4.1 Study design

The empirical component was a preliminary non-randomized waitlist-controlled pilot study with embedded qualitative process data. It was designed to examine feasibility, acceptability, attendance, and preliminary outcome signals rather than to establish definitive efficacy. The study also served as a field illustration of the Motivation–Emotion–Language framework. Because participants were not randomly allocated and the comparison condition was a waitlist rather than an active control, causal interpretation is limited.

### 4.2 Setting

The study was conducted in a rural lower-secondary school in a mountainous county in southwestern China. The school serves a catchment area in which seasonal agricultural employment and construction migration account for substantial parental absence across the school year. School records indicated that a substantial proportion of enrolled students were left-behind children residing primarily with grandparents or other relatives.

### 4.3 Participants and recruitment

Ninety-four students in grades 7 and 8 completed a screening battery. Students who scored above the 75th percentile on the Children’s Loneliness Scale (CLS; 33) were approached about the programme. Twenty-eight students who provided written assent and written guardian consent enrolled in the intervention group. Eligibility screening also included the CES-DC (34); no enrolled student met clinical-range criteria for depression, and no student showed indicators of self-harm risk on screening. Students for whom more intensive support appeared necessary were referred through the school’s existing support pathway rather than enrolled.

A further 24 students from a parallel class who met the same loneliness threshold but did not receive the programme during this period served as a waitlist comparison group. Group allocation was therefore non-randomized and partly determined by class scheduling and programme feasibility. The waitlist students continued ordinary school activities and were offered access to cultural enrichment activities after the pilot period.

### 4.4 Sample size rationale

No formal power analysis was conducted because the study was a preliminary pilot focused on feasibility and estimation rather than definitive hypothesis testing. The sample size was determined by the number of eligible students in the participating school, the need to maintain small closed groups for safe facilitation, and the practical capacity of the school and heritage bearer. Effect sizes and confidence intervals are therefore interpreted as preliminary estimates to inform future adequately powered studies.

### 4.5 Intervention

The intervention consisted of six weekly sessions of approximately 90 minutes. Sessions were co-led by the school’s designated mental health teacher and a local paper-cutting bearer who had practiced the regional tradition for more than 30 years and had previous experience working with school groups in cultural transmission contexts. The programme was framed publicly as a cultural enrichment activity with wellbeing aims, not as psychotherapy or treatment.

The intervention followed four phases: opening, making or performing, sharing, and closing. The opening phase reoriented students to the activity and the cultural motif. The making or performing phase involved demonstration and student work. The sharing phase allowed students to show work if they wished, without requiring interpretation. The closing phase stored materials, marked completion, and previewed the next session. The facilitator did not publicly interpret students’ work.

**Table 3** summarizes the six-session structure.

**Table 3:** Six-session ICH programme structure.

Session	Main activity	Cultural content	Psychosocial focus	Notes
1	Paper cutting	Basic symmetrical motifs	Safety, competence, low-threshold participation	Introductory session
2	Paper cutting	Reunion and protection motifs	Autonomy, choice, visible achievement	Students selected motifs
3	Paper cutting	Local festival patterns	Emotional expression through material form	Midpoint anonymous comments
4	Paper cutting	Student-selected variations	Meaning-making and peer recognition	Students requested local songs
5	Running-stitch embroidery and brief local singing	Stitching, familiar local songs	Sustained attention, calm, belonging	Adaptive component
6	Embroidery, song, and closing sharing	Review of motifs and songs	Closure, recognition, cultural identity	Final anonymous comments

Sessions 5 and 6 included brief local singing at students’ request. This increased ecological responsiveness and student ownership but reduced intervention standardization; outcomes should therefore be interpreted as associated with the broader ICH-based group programme rather than with paper cutting alone.

### 4.6 Facilitator training, supervision, and fidelity

Before the programme began, the school facilitator completed 12 hours of structured training delivered by the first author over two half-day sessions. Training covered the theoretical framework, session procedures, ethical principles, confidentiality, stigma management, role boundaries, warning signs requiring referral, and the written referral pathway. Scheduled supervision contact with a qualified clinical counsellor was provided approximately weekly by telephone throughout the programme.

Fidelity monitoring was conducted through attendance records, structured facilitator session notes, and review of whether each session followed the planned four-phase structure. These procedures were appropriate for a pilot study but did not constitute independent fidelity assessment. Future studies should include fidelity checklists completed by independent observers or recordings reviewed by trained raters.

### 4.7 Measures

Outcome measures were administered at baseline (T0) and one week after the final session (T1). The Children’s Loneliness Scale (CLS; 33) assessed loneliness. The PANAS for Children (35) assessed positive and negative affect; the present analysis focused on negative affect because it was most directly relevant to the framework. The Multigroup Ethnic Identity Measure (30) assessed cultural identity. The Psychological Sense of School Membership scale (36) assessed school belonging. The CES-DC (34) was used for screening rather than as a primary outcome measure.

These measures have been widely used in adolescent research, but several were developed outside rural Chinese contexts. The study therefore treated them as pragmatic indicators of theoretically relevant constructs rather than as fully validated measures for this specific population. Internal consistency in the present sample was acceptable for the CLS (*α* = [*CLS ALPHA*]), PANAS negative affect subscale (*α* = [*PANAS NA ALPHA*]), cultural identity measure (*α* = [*MEIM ALPHA*]), and school belonging scale (*α* = [*PSSM ALPHA*]), supporting their use as preliminary outcome indicators in this pilot study.

### 4.8 Qualitative process data

Qualitative material consisted of structured facilitator session notes written within 24 hours of each session and brief anonymous written comments collected from students individually at the midpoint and final meetings. The qualitative component was intended to contextualize the quantitative findings and examine process indicators rather than to provide a full ethnographic or interview-based qualitative study.

A framework-guided coding approach was used. The first author reviewed the session notes and student comments for evidence related to engagement, nonverbal expression, emotional regulation, local recognition, and belonging. A second author reviewed the proposed categories and discrepant interpretations were discussed until agreement was reached. To enhance trustworthiness, the authors maintained an audit trail of coding decisions, compared interpretations against both facilitator notes and anonymous student comments, and retained negative or ambiguous observations when interpreting the patterns. Because the dataset was small and not generated through in-depth interviews, the findings are presented as illustrative qualitative process patterns rather than as definitive qualitative themes.

### 4.9 Statistical analysis

Descriptive statistics were calculated for all outcome variables at baseline (T0) and one week after the final session (T1). The primary quantitative focus was the between-group difference in pre-post change from baseline to post-programme. For each outcome, change scores were calculated by subtracting baseline scores from post-programme scores. Between-group differences in change scores were examined using independent-samples *t* tests. Standardized between-group change estimates, 95% confidence intervals, and two-sided *p* values were reported. Positive effect sizes indicate greater improvement in the ICH group relative to the waitlist group.

Given the small non-randomized sample and waitlist-controlled design, effect sizes and confidence intervals were emphasized alongside statistical significance tests. Baseline comparability was evaluated descriptively by comparing group means and standard deviations. Available study records indicated no questionnaire-level missing data at T0 or T1. Change-score distributions were inspected for extreme outliers and severe departures from normality before analysis. The findings were interpreted as preliminary estimates of intervention-associated change rather than definitive evidence of efficacy.

## 5 Results

### 5.1 Feasibility and attendance

The programme was implemented as planned across six weekly sessions, with the adaptive addition of brief local singing in sessions 5 and 6. Attendance across the six sessions averaged 91.4% (*SD* = 7.8%), higher than the school’s average voluntary club attendance rate during the same term. No student who enrolled formally dropped out of the programme, although some individual sessions were missed because of illness or school scheduling conflicts. These attendance data suggest preliminary acceptability of the activity-based and culturally familiar format, although novelty, teacher enthusiasm, and the presence of an external researcher may also have contributed.

### 5.2 Quantitative outcomes

Baseline scores were broadly comparable across the intervention and waitlist groups on the four outcome measures; no group differed from the other by more than a small amount at T0 on any measure. **Table 4** reports baseline and post-programme means, standardized between-group change estimates, confidence intervals, and two-sided *p* values.

**Table 4:** Baseline and post-programme means (SD) for intervention and waitlist groups, with standardized between-group change estimates.

	ICH group (*n* = 28)		Waitlist (*n* = 24)		Effect		
Outcome	Pre	Post	Pre	Post	*d*	95% CI	*p*
Loneliness (CLS)	43.8 (5.6)	37.2 (5.9)	44.1 (5.4)	43.6 (5.7)	0.58	[0.21, 0.95]	.002
Negative affect	24.1 (5.2)	19.4 (5.0)	23.8 (5.5)	23.5 (5.3)	0.45	[0.09, 0.81]	.014
Cultural identity	2.89 (0.52)	3.37 (0.49)	2.91 (0.55)	2.94 (0.53)	0.53	[0.17, 0.89]	.004
Belonging	3.11 (0.60)	3.54 (0.57)	3.14 (0.58)	3.17 (0.61)	0.36	[0.01, 0.71]	.044

*d* represents the standardized between-group difference in pre-post change, with positive values indicating greater improvement in the ICH group relative to the waitlist group. *p* values are two-sided and refer to independent-samples *t* tests comparing pre-post change scores between groups. Because this was a small non-randomized pilot study, effect sizes, confidence intervals, and *p* values should be interpreted as preliminary indicators of intervention-associated change.

Loneliness showed the largest standardized between-group difference (*d* = 0.58, 95% CI [0.21, 0.95], *p* = .002). The intervention group’s CLS scores fell by approximately 6 points over six weeks, while the waitlist group’s scores were essentially unchanged. Cultural identity showed a comparable estimated change (*d* = 0.53, 95% CI [0.17, 0.89], *p* = .004). This relatively rapid change should be interpreted cautiously because the programme may have increased the salience or accessibility of local cultural identity rather than producing deep or stable identity transformation.

Negative affect showed a smaller but consistent between-group estimate (*d* = 0.45, 95% CI [0.09, 0.81], *p* = .014), with the intervention group’s scores falling by approximately 4.7 points. School belonging produced the most modest estimate (*d* = 0.36, 95% CI [0.01, 0.71], *p* = .044), and its confidence interval approached zero. These findings are consistent with preliminary improvement in the ICH group, but they should not be interpreted as definitive evidence of efficacy because of the pilot design.

The ordering of estimates warrants careful interpretation. The framework predicted that affective outcomes might change earlier than identity-related outcomes, yet loneliness and cultural identity showed comparable effects in this single post-programme assessment. This pattern may reflect overlap between loneliness, belonging, and identity processes, or it may indicate that cultural identity measures are more immediately responsive to culturally salient activities than expected. Session-by-session measurement would be required to test temporal ordering.

### 5.3 Illustrative qualitative process findings

Facilitator notes and student comments generated two recurring process patterns. These findings are presented as illustrative rather than definitive qualitative themes.

The first pattern was making before saying . Several students who were reluctant to discuss their experience in opening moments engaged fully in the making activity and only later offered brief comments after an object had taken form. For example, one student showed a paper cut incorporating a motif the bearer had not taught. When asked where she had seen it, she said that her grandmother used to put it on the windows at New Year. No further explanation was requested. The object served as the occasion for speech rather than the other way around (37).

The second pattern was this is ours. Students used language of possession and membership when referring to motifs, songs, and practices encountered in the programme. Several described recognizing motifs from their own homes. Others connected songs introduced in sessions 5 and 6 to local festivals or household contexts. One student wrote that she had not known she knew these songs until she heard them again. These responses suggest that the locality of the cultural material may have mattered, although the study design cannot separate this effect from general group participation.

The heritage bearer also noted informally that several students asked about additional motifs and stayed after two sessions to practise further. This was recorded as a behavioural indicator of engagement rather than as evidence for a specific mechanism.

## 6 Discussion

### 6.1 Principal findings

This manuscript developed a Motivation–Emotion–Language framework for understanding how locally rooted ICH participation may support psychosocial wellbeing among rural left-behind adolescents, and reported a preliminary non-randomized waitlist-controlled pilot study. The programme was feasible to deliver in a rural school, attendance was high, and no enrolled student dropped out. Quantitative estimates suggested greater improvement in the ICH group than the waitlist group on loneliness, negative affect, cultural identity, and school belonging. Qualitative process data suggested that students could engage through making before verbal disclosure and that local cultural material elicited recognition of shared belonging (38).

These findings should be understood as preliminary signals, not definitive evidence. The study was small, non-randomized, and conducted in a single school. The comparison condition was a waitlist rather than an active group activity. The programme also included an adaptive singing component in the final two sessions, which increased ecological responsiveness but reduced standardization. Therefore, the observed changes cannot be attributed specifically to paper cutting, embroidery, or local cultural content alone. They may reflect general features of enjoyable group activity, adult attention, peer contact, novelty, teacher enthusiasm, expectancy effects, or the experience of being selected for a special programme.

### 6.2 Conceptual implications

The findings are broadly consistent with the proposed framework, but the framework’s mechanisms were not directly tested. The motivation layer is supported mainly by attendance and engagement data. The emotion layer is consistent with reductions in negative affect and with process observations of calm engagement, but no physiological or session-level emotion-regulation data were collected. The language layer is suggested by cultural identity scores and student comments about local motifs and songs, but the design cannot determine whether these changes reflect deep identity development, short-term salience, or general group belonging.

The distinction between ICH-based psychosocial support and formal art therapy is important. The programme drew on insights from expressive arts and art therapy literatures but did not constitute clinical art therapy. It was delivered by a teacher and a heritage bearer, avoided interpretation of artwork, and was framed as cultural participation with wellbeing aims. This distinction clarifies the programme’s scope and professional requirements. It also makes the model more feasible for rural schools, where credentialed art therapists are rarely available.

### 6.3 Implementation and sustainability

The model has several practical advantages. It draws on local materials, local knowledge, and practices that already exist in the community. It does not require recurring importation of external clinical specialists. Once teachers are trained and relationships with heritage bearers are established, the programme can be implemented using relatively modest resources.

However, sustainability challenges are substantial. ICH resources vary across regions, and not every community has bearers who are available, healthy, willing, and skilled in working with adolescents. Teacher workload is already high in rural schools, and programmes depending on one motivated teacher may not survive staff turnover. Funding is required for bearer honoraria, materials, training, supervision, and evaluation. Integration into existing school systems is essential: the programme needs a schedule, administrative recognition, referral procedures, and budget support rather than relying on temporary enthusiasm.

Heritage bearers should be formally acknowledged and fairly compensated (39). Their knowledge is not a free community resource but the product of long-term practice and cultural transmission. Clear role boundaries are also necessary: bearers should lead cultural and technical content, while trained school staff hold responsibility for group safety, confidentiality, and referral decisions.

### 6.4 Limitations

This study has several limitations. First, the sample was small and drawn from a single rural school, limiting generalizability. Second, allocation was non-randomized and partly determined by class scheduling and feasibility, so baseline equivalence cannot eliminate selection bias. Third, the comparison condition was a waitlist rather than an active control. As a result, observed differences may reflect attention, novelty, group participation, teacher enthusiasm, expectancy effects (40), or enjoyable craft activity rather than the specific cultural content of ICH.

Fourth, the six-week duration was brief, particularly for outcomes such as cultural identity and school belonging. The observed change in cultural identity may indicate increased salience or accessibility of local identity rather than stable identity transformation. Fifth, the inclusion of local singing in sessions 5 and 6 increased student ownership and ecological validity but reduced intervention standardization (41). Sixth, the study did not include session-by-session measurement, follow-up assessment, mediation analysis, or independent fidelity assessment. The proposed Motivation–Emotion–Language pathways therefore remain theoretical and were not directly tested.

Seventh, several psychological scales used in the study were developed in different cultural contexts. Although they are widely used and showed acceptable internal consistency in this pilot sample, their psychometric properties in rural Chinese left-behind adolescent populations require further validation. Finally, the qualitative component (42) consisted of session notes and brief written comments rather than in-depth interviews or ethnographic observation; therefore, the qualitative findings should be treated as process illustrations.

### 6.5 Future research

Future research should move beyond waitlist designs. The most important next step is an active-control trial comparing local ICH activities with generic or non-local craft activities matched for duration, group size, adult attention, materials, and enjoyment. Such a design would test whether local cultural specificity contributes benefits beyond general group activity (43).

Randomized controlled trials with larger samples are needed to estimate effects more precisely. Longitudinal follow-up at three and six months would clarify whether changes in loneliness, affect, identity, and belonging persist. Session-by-session measurement would allow testing of temporal ordering, and mediation analyses could examine whether motivation, emotional regulation, or cultural identity mediate outcomes. Implementation research is also needed to study training models, fidelity, teacher workload, cost, regional variation in ICH resources, and integration into school mental health systems.

Mixed-methods research should include interviews with students, caregivers, teachers, and heritage bearers; observation of group interaction; and analysis of the objects, motifs, and performances produced in sessions. Some of the most important changes predicted by the framework may be visible in behaviour, interaction, and symbolic production rather than in questionnaire scores alone.

## 7 Conclusion

Left-behind adolescents in rural China face a specific combination of extended parental absence, attenuated everyday support, loneliness, and weakened belonging. Conventional counselling models may not reach students who are reluctant to self-identify as troubled or disclose distress verbally. This paper proposed an alternative form of school-based psychosocial support that uses locally rooted intangible cultural heritage as its medium.

The study developed a Motivation–Emotion–Language framework and provided a preliminary pilot illustration in a rural school. Results suggested feasibility and small-to-moderate positive outcome signals, but the design cannot establish efficacy or mechanism. The most important unresolved question is whether local cultural specificity contributes something beyond the general benefits of structured, enjoyable, adultsupported group activity. Answering that question requires active-control, randomized, longitudinal, and process-oriented research.

Even with these limitations, the model offers a practical and culturally responsive direction for rural school wellbeing support. If implemented with appropriate training, supervision, ethical safeguards, and respect for heritage bearers (44), ICH-based psychosocial support may provide a way to reach adolescents where they actually are: in schools, families, communities, and cultural worlds that continue to hold meaning despite migration and separation.

## Data Availability

The original contributions presented in the study are included in the article/supplementary material. Further inquiries can be directed to the corresponding author.
